# Order Reduction of the Chemical Master Equation via Balanced Realisation

**DOI:** 10.1371/journal.pone.0103521

**Published:** 2014-08-14

**Authors:** Fernando López-Caamal, Tatiana T. Marquez-Lago

**Affiliations:** Integrative Systems Biology Unit, Okinawa Institute of Science and Technology, Kunigami, Okinawa, Japan; Technische Universität Dresden, Medical Faculty, Germany

## Abstract

We consider a Markov process in continuous time with a finite number of discrete states. The time-dependent probabilities of being in any state of the Markov chain are governed by a set of ordinary differential equations, whose dimension might be large even for trivial systems. Here, we derive a reduced ODE set that accurately approximates the probabilities of subspaces of interest with a known error bound. Our methodology is based on model reduction by balanced truncation and can be considerably more computationally efficient than solving the chemical master equation directly. We show the applicability of our method by analysing stochastic chemical reactions. First, we obtain a reduced order model for the infinitesimal generator of a Markov chain that models a reversible, monomolecular reaction. Later, we obtain a reduced order model for a catalytic conversion of substrate to a product (a so-called Michaelis-Menten mechanism), and compare its dynamics with a rapid equilibrium approximation method. For this example, we highlight the savings on the computational load obtained by means of the reduced-order model. Furthermore, we revisit the substrate catalytic conversion by obtaining a lower-order model that approximates the probability of having predefined ranges of product molecules. In such an example, we obtain an approximation of the output of a model with 5151 states by a reduced model with 16 states. Finally, we obtain a reduced-order model of the Brusselator.

## Introduction

Markov chains are dynamical systems that model a broad spectrum of physical, biological, and engineering systems. Along with their broad range of applications, one of the main advantages of Markov chains is that some of them can be easily handled as time-invariant, linear systems [1]–[3].

In this paper, we focus on continuous-time, discrete-state, homogeneous, irreducible Markov chains with a finite number of states. The probability of being in any state is governed by a set of linear ordinary differential equations (ODEs), where individual ODEs correspond to each state of the system, describing all possible transitions in and out of such states. This set of ODEs is commonly referred to as forward Kolmogorov equation or chemical master equation and might have large dimensions even for simple systems. Hence, obtaining a solution for such a system might be analytically intractable and computationally demanding.

Provided that one is interested only in some states or a combination of states of the Markov chain, it is possible to obtain a reduced order model via the balanced realisation of the linear system that describes the probability of being in such states. The reduced model has a smaller number of coupled differential equations, yet approximates the output of the full model with an error bound proportional to the sum of the Hankel singular values neglected to obtain the reduced model [4]–[7]. Given chemical reaction networks in a homogeneous media and in thermodynamic equilibrium can be described as Markov chains, it is possible to apply our methodology to this class of systems.

It is worth noting that there exist alternative approaches to obtain reduced order models from the CME. For instance, in [8] the author analysed methods to approximate the solution of selected states of the CME, when such solutions can be expressed as the product of two probability density functions: one that describes probabilities of states of interest and a second that depends on the rest of states. This latter probability distribution can be approximated by its mean, for instance, so as to yield an approximated probability density function for those probabilities of interest. However, this approach might yield coarse results if the underlying assumptions are crude.

Other approaches make use of Krylov subspaces to approximate the solution of the exponential matrix that generates the solutions of the Markov chain [9], [10]. Additionally, when the species can be classified by its behaviour into stochastic or deterministic, the authors in [11] propose a methodology in which the CME can be solved directly and efficiently, when the number of species with stochastic behaviour is low. In this direction, works like [12] avail of a time scale separation to estimate the solution of the fast-varying species; and use this estimation to approximate the trajectories of the slow-varying species.

As an alternative, when the analytical or computational treatment of the Markov chain is infeasible, it is common to opt for numerical simulations of the stochastic system and analyse the outcome statistically. References [13] and [14], among many others, provide surveys of simulation methods of stochastic reaction networks. However, these methods might require large computational times to yield accurate results.

A different way to reduce CMEs is to consider subsystems that focus on features of interest. From the chemical perspective, in [15] the authors showed as a proof-of-concept that the simple reaction 
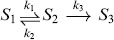
 can only be accurately represented by 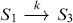 under special conditions on the parameters 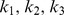, which render the dynamics of the species 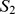 irrelevant for the behaviour of 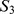. This study highlights the shortcomings of neglecting species within a stochastic reaction network. In this paper, we adopt a different approach and overcome these difficulties by deriving a reduced-order model. Such reduced-order model accurately approximates the dynamics of the underlying Markov chain for selected states with any kind of reaction propensities.

In some cases, the dynamics of the species population with stochastic behaviour can be expressed as the weighted sum of the species population given by the deterministic framework and a random variable, which represents the stochastic behaviour of the modelled system. Under these circumstances, an associated Fokker-Plank equation might be derived from which different stochastic traits may be analysed. By using this approach, one might obtain an ODE whose dimension is typically smaller than the dimension of the corresponding CME. This approach is know as *van Kampen's system size expansion* and a more precise explanation can be found in [3]. However, any approach based on van Kampen's system size expansion suffers from such restricted applicability, since they hinge on the ability of expressing random variables as an explicit sum of a deterministic variable and a random one.

There exist, however, exact approaches that abridge specific topologies of reaction networks. For instance, in [16], [17] different classes of monomolecular reaction networks are exactly represented as reactions characterised by delay distributions. In turn, works like [18], [19] are committed to obtain exact analytical solutions of stochastic chemical reaction networks with linear and nonlinear reactions. Importantly, once a reduced ODE set via balanced realisation is obtained, one can avail of the results in ([18], Sec. 2.2) to derive a closed-form expression for approximation of the CME solution.

We illustrate our methodology with the analysis of a reversible, stochastic reaction whose CME has 301 states. In contrast, an adequate reduced order model has only 10 states and yield an 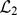 gain of the approximation error of 587.91×10^−6^. Later, we obtain a reduced order model that approximates the catalysed conversion of a substrate to a product, even in cases in which a rapid equilibrium approximation fails to obtain accurate results [20], [21]. In contrast to the approaches in [20] and references therein, we do not assume any particular relation among the parameters and initial conditions. Hence, our methodology is more widely applicable. For such a system, the simulation of the reduced model may be several orders of magnitude faster than the simulation of the CME. However, there exist an initial cost in computational time to derive the reduced order model. Thus, obtaining a reduced model is profitable when the lower-dimensional ODE set is used repeatedly. In addition, we derive a model that approximates the probability of having predefined ranges of product molecules, in the same catalytic substrate conversion. To finalise we derive a reduced order model of the Brusselator.

## Analysis

### Continuous-Time, Discrete-State, Homogeneous Markov Chain

Consider a discrete and finite set of states
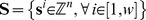
(1)and let the system's state at time *t* be denoted by 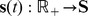. Moreover, we consider that the transition from one state to another can be modelled by a time-homogeneous Markov chain, i.e., the next state, 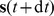, only depends on the current state, 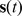, independently of *t*. We use 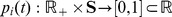 to denote the probability, 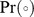, of the system's state to be 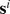 at time *t*. This notation and the Markov property add up to



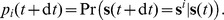



We gather the probabilities for every state in the column vector
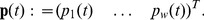
(2)


Let us denote the transition probability from the initial state *j* to state *i* at time 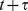 by 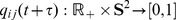. That is to say,
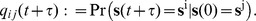



The time-homogeneity property of the Markov chain implies
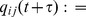


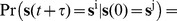


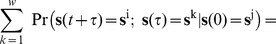


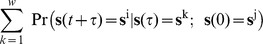


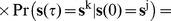


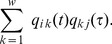
(3)


In matrix form (3), known as *Chapman-Kolmogorov equation*, is
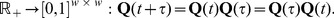
(4)


This matrix gathers all the transition probabilities as a function of time and, by consequence, its columns add to one for all *t*. Additionally, if the Markov chain is *irreducible*, 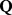 has a simple eigenvalue 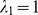, and 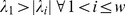. This is a consequence of the Perron-Frobenius Theorem as described in ([2], Ch. 6), for example. In the rest of this paper we deal with finite, irreducible, homogeneous, continuous-time, discrete-state Markov chains exclusively.

Our main interest is to determine the time-dependent probabilities of being in any state of the chain. To this end, we consider the *infinitesimal generator* of the Markov chain defined as
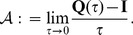
(5)


The elements of the matrix above are
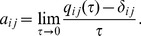
(6)


Here 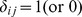, when 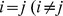, respectively). From (6) it can be shown that the elements 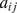 satisfy
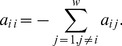
(7)


The last relationship above shows that every column of 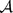 adds up to zero, provided each column of 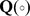 adds up to one.

It is well-known that 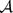 is the generator of the positive semigroup that governs the evolution of **p**(*t*) (see [22], Sec. 5.6, for instance):
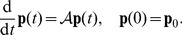
(8)


Under our assumptions, the Markov chain is irreducible and with a finite number of states. Hence, 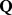 has a unique Frobenius eigenvalue with algebraic multiplicity one. The simple Perron-Frobenius eigenvalue of the stochastic matrix 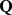 in (4) is 1 [23]. Now, let 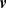 and 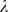 be the right Perron-Frobenius eigenvector and eigenvalues of 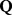, then the eigenvalues of 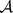 Satisfy
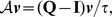


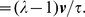



That is, 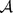 preserves the configuration of the eigenvalues of 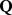, upon shifting one unit to the left and rescaling. This implies that 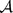 has a zero eigenvalue and the rest of its eigenvalues have negative, real parts, as confirmed by analysing the Geršgorin circles of the columns of 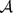. We refer the interested reader to Appendix S1 for a proof of this statement.

Note that the dimension of 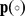, 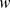, might be large as it represents all the configurations of a system with 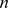 characteristics. In the population and biochemical contexts, 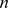 represents the number of species, whereas 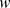 denotes the number of all the possible combination of species' population counts. In the following section, we model a stochastic chemical reaction network with the Markov chains described above.

### Chemical Master Equation

Now, let us consider 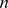 species in a homogeneous medium and in thermodynamic equilibrium and a set of 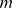 reactions represented by
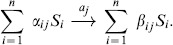
(9)


Let the entries of the stoichiometric matrix 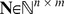 be
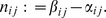



Furthermore, let us consider a vector comprised of the number of molecules, 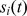, for every species, 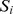:
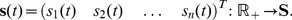
(10)


The finite set 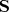 above was defined in (1) and contains, at least, all the possible combinations of the species' molecular numbers in the reaction network. Consider that the *i^th^* reaction is the only reaction happening within the interval 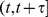. Hence the number of molecules at time 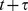 is
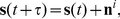
(11)where 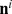 represents the *i^th^* column of 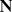.

This reaction network may be modelled by the continuous-time, discrete-state jump Markov process described previously. The states of the Markov chain are the elements in 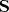. In turn, the vector 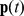 in (2) gathers the time-dependent probabilities of being in every state, whose time evolution is governed by (8). Additionally, the stochastic behaviour of thermally stable and spatially homogeneous reaction networks has been described in [24]. Based on such a work, Table 1 summarises the transition rates between states of the system.

**Table 1:** Reactions and their propensity function.

Reaction	Propensity
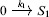	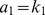
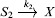	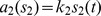
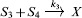	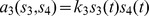
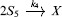	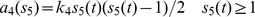

The symbol 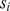 denotes the number of molecules of the species 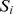. The symbol 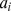 denotes the propensity of reaction 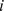.

To construct the matrix 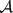 in (8), we have to evaluate the probabilities' transition rate for all states 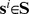 and arrange them as the entries of 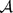 as follows
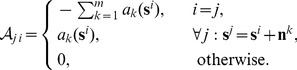
(12)


In the next section, we present a methodology used to obtain reduced order models, which are capable of reproducing the dynamical behaviour of a linear system with a smaller number of ODEs.

### Balanced Model Reduction

In this section, we present an overview of a methodology used for obtaining lower dimensional models via balanced realisation. The literature on this topic is vast and we refer the interested reader to [5]–[7], [25] for a comprehensive presentation of this type of model reduction.

Let us consider a linear system of the form
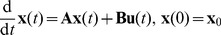
(13a)

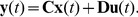
(13b)


Here 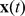 represents the state of the system; 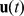 is the forcing term of the differential equation (13a); and 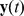 comprises the variables of interest expressed as a linear combination of 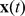. We will assume that all the eigenvalues of 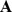 have negative real parts, i.e. 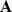 is stable, and that the system in (13) is both controllable and observable. These last two properties are commonly used in the control-theory literature and their definitions are given below.

*Controllability* is the property of (13) which ensures that it is possible to steer the state of the system from any initial condition 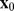 to any desired state at a specific time, by the application of an adequate forcing function 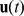. In turn, *observability* refers to the capability of computing 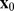 given the knowledge of 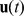 and 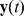 for all previous time 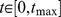.

The definitions of both controllability and observability just express that systems with such properties are capable of being steered to a desired state, and that 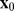 can be computed from the history of 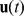 and 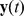, respectively. These definitions, however, are not constructive, in the sense that they do not explain how one can design 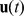 or obtain 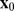. These topics are beyond the scope of this paper, but the interested readers can find further details in [26].

Now, controllability and observability hold when 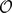 and 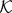, the controllability and observability matrices, respectively, are full rank
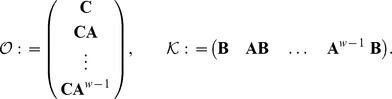



When (13) is simultaneously stable, observable, and controllable there exist unique, symmetric, positive-definite matrices 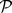 and 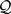 which are solution of the following Lyapunov equations
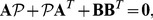
(14a)

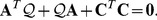
(14b)


The singular values of the product of 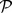 and 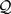 are known as the Hankel singular values, 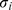, of the system.

Now, the linear system in (13) can be expressed in different coordinates than 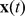. That is to say, if we prefer to use the coordinates
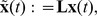
where 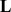 is a square, full-rank matrix of appropriate dimensions. Then, we can rewrite the linear ODE in (13) as



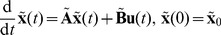
(15a)

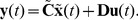
(15b)


Here, 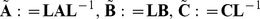. We note that for both systems (13) and (15) the function 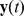 is exactly the same, under the application of the same forcing function 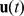. The reason for considering these alternative coordinates 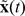 is that there exists a 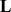, such that the solutions of the corresponding Lyapunov equations in the coordinates 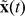 have the following property
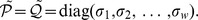



Here 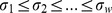. When this condition is satisfied, the system (15) is Lyapunov balanced [4]. The details on the construction of 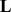 are given in [4] and algorithms for the derivation of Lyapunov balanced realisations have been implemented in Python and Matlab, among others.

If the original system (13) is not stable nor represented by its minimal realisation (i.e. simultaneously observable and controllable), we suggest the transformation in [27] to obtain its Lyapunov balanced form.

An advantage of having a balanced realisation is that the magnitude of the singular values 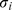 decays quickly as *i* increases. There are several techniques that avail of this observation to derive reduced order models, depending on the required characteristics of such a reduced model [6].

Let us consider that 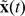 are the coordinates of the Lyapunov balanced realisation. One of the simplest approach to obtain a reduced order model is to partition 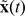, to obtain
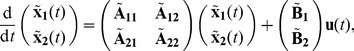


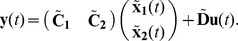



Here, 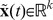 and the rest of the vectors and matrices have the appropriate dimensions. This separation also induces the partition 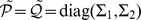, where 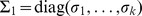 and 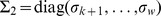. By neglecting the states 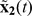 associated to the small Hankel singular values, the truncated model becomes
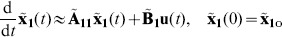
(16a)

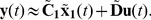
(16b)


This model is known to preserve the most important eigenvalues of the original system, and interested readers can find more details in [28]. However, some other properties such as steady state are slightly modified. When such a property is of interest, *model reduction by residualisation* is more suitable [7]. Both of these methods are already included in languages such as Python and Matlab, where the balanced realisation of a linear system is in the function *balreal* and the model reduction via truncation and residualisation is in *modred*.

It is also important to mention that error of approximation satisfies the following bound
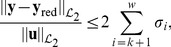
(17)where 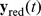 is the output of the reduced model. In the following, we refer to this bound as the 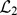 *gain of the approximation error*, where the term *gain* refers to the fact that we are considering the size of error of approximation normalised by the size of the forcing function 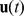. Please, refer to Appendix S1 for the definition of 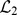 norms and for a derivation of such an error bound. The expression in (17) suggests a trade-off between the accuracy of the approximation and the size of the reduced order model. To see this, notice that 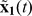 has 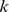 elements. As 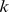 grows, the error bound in (17) will decrease, at the cost of obtaining a larger reduced model in (16). A good initial guess for the magnitude of 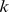 is obtained by neglecting those states associated to Hankel singular values which are three orders of magnitudes smaller than the largest one; namely, by finding a 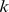 such that 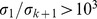.

In summary, given an arbitrary system of the form (13), the first step for obtaining a reduced model is to find a coordinate transformation 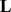 that expresses (13) in its Lyapunov balanced form. At a second step, we have to determine the size of the appropriated reduced model. To do so, we consider a partition of the state of the system expressed in the coordinates 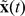. The advantage of using these coordinates is that the state 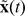 is organised such that the entries are progressively less relevant with respect to 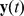. Hence, by neglecting the last entries of 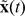, denoted as 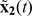, we can obtain reduced order models that approximate the behaviour of the full one. In the forthcoming section, we build upon the material in this section to obtain a reduced order model of the representation of a continuous-time, discrete-state, homogeneous, irreducible Markov chain.

### Order Reduction of Infinitesimal Generators

In this section we are interested in the probability of being in some (linear combination of) states of the Markov chain, which we denote as 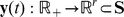. As noted in Equation (8), the vector 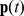 evolves according to the linear ODE
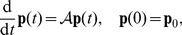
(18a)

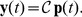
(18b)


Also, as was mentioned previously, the infinitesimal generator 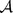 of an irreducible Markov chain with finite states has the properties:
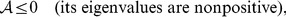
(19a)

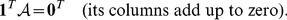
(19b)


Without loss of generality, we will assume that the system (18) is both controllable and observable. However, it is worth noting that whenever it lacks any of these two properties, there always exist a transformation that obtains the observable and controllable subspace of (18); namely, the Kalman Decomposition [26], [29].

To consider a reduced model that does not have a zero eigenvalue, let 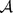 be partitioned as follows:
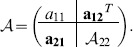
(20)


Here 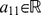, 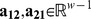, and 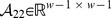. This partition is considered to ensure conformability of the product with the following similarity transformation
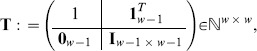
(21a)

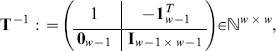
(21b)

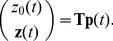
(21c)


The coordinate transformation 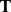 above is constructed such that 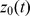 in (21c) is a state that represents the sum of all the entries of the probability vector 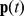. As we confirm below, this state has a constant value equal to one for every time. Furthermore, the matrix 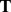 in (21a) implies that the entries of 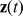 in (21c) are 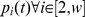. By differentiating (21c) and using the expressions in (18), we get
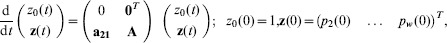
(22)

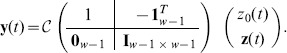



The solution for the first state is the unitary step function, that is 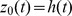. By substituting this solution in the ODE above, we have
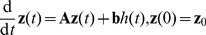
(23a)

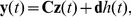
(23b)where



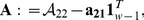
(24a)

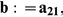
(24b)

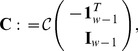
(24c)

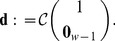
(24d)


The spectrum of 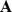 in (23) has all the eigenvalues of 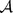, except for the zero eigenvalue. To see this, recall that the trace of a matrix is the sum of its eigenvalues. As (22) arises from a similarity transformation applied to (18), we have that
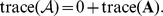



Under our assumptions, 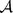 has only one zero eigenvalue and, hence, the spectrum of 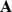 is composed by the nonzero eigenvalues of 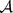. All these eigenvalues have negative real parts.

As mentioned earlier, although the triplet 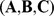 in (23) might not be a minimal realisation, it is always possible to obtain a model which is both controllable and observable via its Kalman decomposition [26], [29]. In fact, the command *balreal* of Matlab's Control System Toolbox will obtain the controllable and observable system before obtaining the balanced realisation. Hence, it is not absolutely necessary to test for these properties separately, when using this software. Thus, for stable systems, we can perform the model balancing to obtain a reduced-order model of the form (16).

So far we had considered that the number of states, 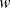, of the Markov chain is finite. However, when considering chemical reaction networks, it is possible to use an approximation of the set of possibles states 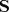, to obtain an ODE set analogous to (18) with the most representative, finite number of states. Due to its approximate nature, the set of ODEs obtained via this truncation of the state space might not present the properties in (19) (see [8], Sec. 2.3 and references therein). Hence the change of variables in (21) would no longer be necessary and a balanced model reduction can be applied directly to set of ODEs arising from such a state space truncation.

Although the lower-dimensional model can be used for obtaining an approximated numerical solution for the probabilities of interest, we would like to notice that one may use the results in ([18], Sec. 2.2) to derive closed-form expressions for these probabilities. In the following section, we study some case studies to show the applicability of these methods.

## Results

In this section, we show the derivation and application of reduced order models, through different examples. We will first analyse one monomolecular reaction and obtain an accurate approximation for the probability of having the conversion of all the molecules from the first species to the second one. Later we derive reduced order models capable of approximating a catalytic conversion of a substrate even in cases in which a rapid equilibrium approximation cannot yield accurate results [20], [21]. Subsequently, we revisit the catalytic substrate conversion to derive the probability of having ranges of product molecules. Finally, we obtain a reduced order model for the Brusselator. In all case studies, we used a 3.2 GHz Quad-Core Intel Xeon computer with 16GB of RAM. Our script was coded in MATLAB

© R2012b.

### Monomolecular Reaction Network

Let us consider the reversible reaction
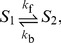
(25)along with the vector composed of species' molecular number 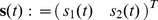. Furthermore, consider an initial number of molecules 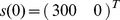. Note that in the reaction above the number of molecules remains constant and equal to the initial 300 molecules. Hence the set of 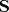 has 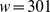 elements and may be ordered as follows



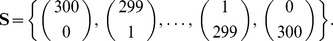



Now, we are interested in the time-dependent probability of having 300 molecules of 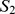, i.e., to be in state 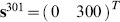. With this formulation, the matrix 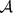 in (18) becomes
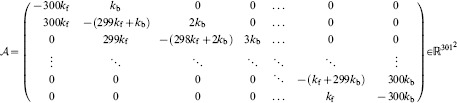
(26)


In turn 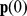 and 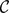 are given by
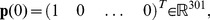
(27a)

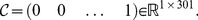
(27b)


**Figure 1:**
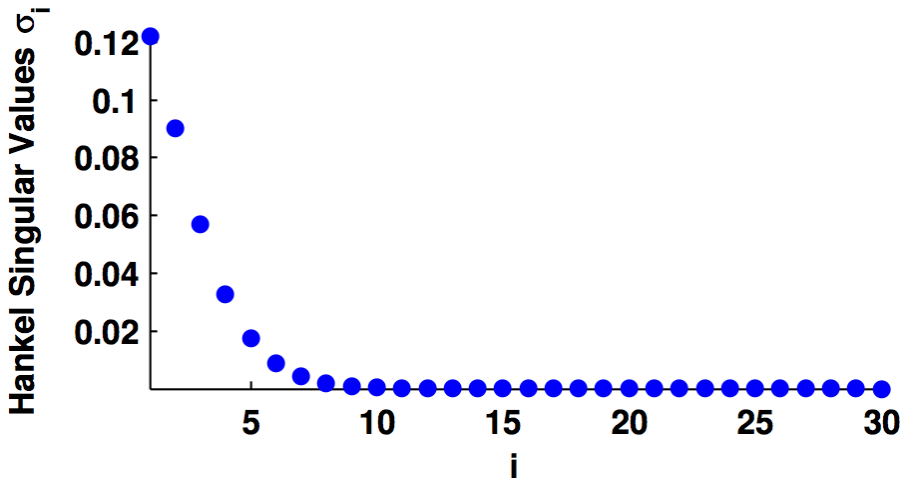
Largest Hankel singular values of the balanced realisation of the model in form of (23), where 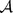 and 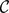 are defined in (26) and (27b), respectively. Additionally 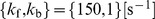.

With the definitions for 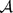, 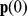, and 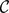 in (26) and (27), respectively, and by choosing the parameters 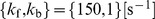, we implemented the model in (23) in Matlab 2012b and obtained its balanced realisation with the command *balreal*. Figure 1 shows the largest 30 Hankel singular values of the balanced realisation's grammian. We observe that the first ten singular values have a large norm in comparison to the rest. By using the command *modred*, we obtained the reduced order model with different number of states; hence, achieving different degrees of approximation.

**Figure 2:**
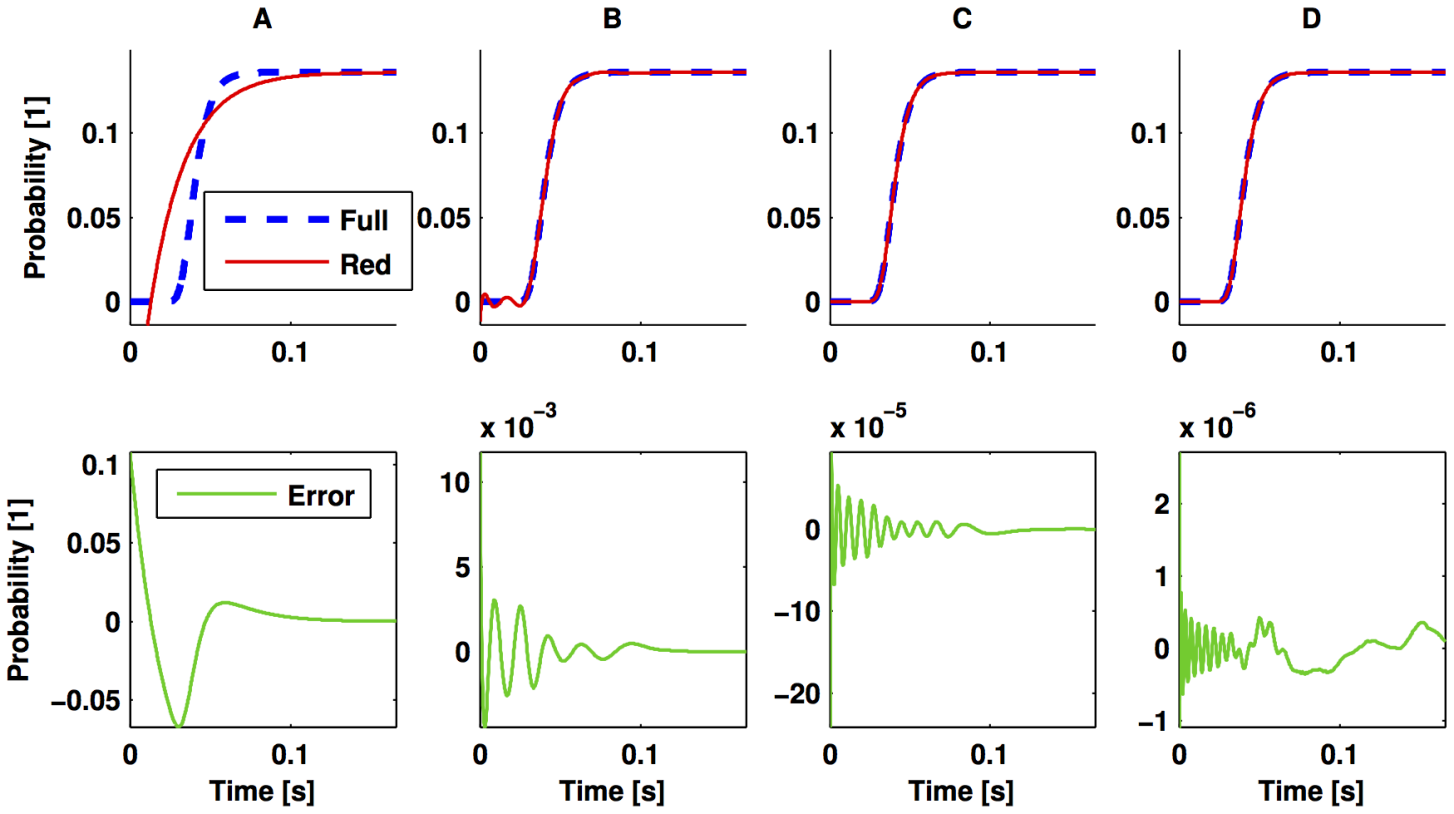
Output comparison of the full CME and the reduced order model. The upper panels depict of the probability of having all the molecules of 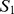 converted to 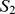 by means of the reversible reaction (25). The discontinuous line represents this probability as obtained with the full model and the continuous lines, those obtained with the reduced order model via balanced realisation. In turn, the lower panels show the difference of full model output and that of the reduced order model. The order of the lower-dimensional model for columns **A, B, C**, and **D** are 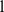, 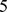, 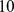, and 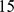 states, respectively. The parameters used for simulations are as in Figure 1.

We depict the impact of the number of states on the error of approximation, in Figure 2. There, we note that a very coarse approximation is achieved when we try to approximate the full model with 301 states by a model of only 1 state (see the lower panel of Figure 2**A**). In turn, when the reduced order model has 10 states, the error of approximation is of order 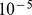, as depicted in the lower panel of Figure 2**C**. Furthermore, if the reduced model has 15 states, the approximation error might already range in the order of the numerical-algorithm integration error, as suggested by the irregular fluctuations shown in the lower panel of Figure 2**D**.

To finalise this section, we note that the 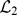 gain of the approximation error is 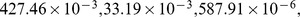 and 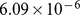, for the reduced models with 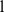, 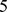, 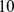, and 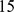 states, respectively. These bounds were obtained by evaluating Expression (17). It should be noted that this is a theoretical bound and does not account for numerical errors during the integration or computation of the Hankel singular values.

In the forthcoming section, we obtain reduced order models for a catalytic substrate conversion, and assess the computational burden required to obtain the reduced order model. In addition, we benchmark the time required for solving numerically the reduced order model against both the computational load required to solve the full order model and the Stochastic Simulation Algorithm (SSA).

### Michaelis-Menten Mechanism

In this section, we consider the reaction network
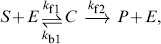
(28)which represents the conversion of a substrate, 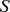, to a product, 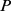, mediated by a catalytic agent, 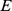, which binds to the substrate to form the complex 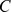. In the deterministic case, it is common practice to approximate the mass-action-based reaction network in (28) via the reaction
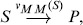
(29)with the following nonlinear reaction rate
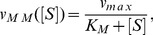
(30a)where 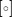 stands for concentration of the argument and



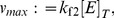
(30b)

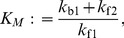
(30c)

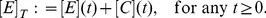
(30d)


The parameter 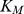 is know as the Michaelis-Menten constant. In [30], it was shown that the dynamics of (28) can be reasonably approximated by (29) when
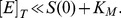
(31)


However, for cases in which the reactions in (28) are better described by a stochastic model, it is still possible to represent the dynamics of 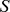 and 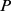 with a reaction of the form (29) by using the propensity
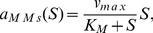
(32)where 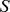 now represents the number of molecules of the substrate per unit volume; 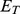 is the total number of molecules of free and bounded enzymes per unit volume; and 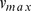, 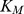 are those of the stochastic model. This representation is valid under the condition



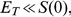
(33)as mentioned in [20]. The basis for this proof is that the condition above induces a time-scale separation which leads some species to converge quickly to their equilibrium. In the following, we refer to this property as the *rapid equilibrium approximation*. Of note, the condition in (33) is not the only one that may allow us to use a propensity of the form of (32); however, we will limit our attention to this condition.

We refer the interested reader to [21] for a rigorous analysis of the validity of propensity of reaction (32). There, the authors compared the variance of fluctuations around the steady-state obtained via the Linear Noise Approximation and the one obtained from the CME with elementary reactions.

We now obtain a reduced order model that approximates the probability of being in selected states of the underlying Markov chain. We derive this reduced model by means of the procedure described in the Analysis section. In contrast to the approaches in [20] and references therein, we do not assume any particular relation among the parameters and initial conditions. Hence, our methodology is more widely applicable. As mentioned earlier, our methodology obtains a small ODE set that approximates the solution of the original CME. In contrast, the approach in [15] is committed to accurately representing a particular reaction network via a single reaction, for specific ranges of parameter values.

Another difference from the approaches in [20] is that they prove the applicability of SSA algorithms with the propensity in (32). In contrast, we derive a dynamical system that approximates the solution of the CME with an *a priori* error bound given by (17). We recall that in the limit, the probability distribution obtained from the SSA trajectories will converge to the solution of the CME. However, depending on the kinetic parameters and network analysed, the SSA might require large computational times to provide results with the desired accuracy.

It is worth noting that even when (28) cannot be represented by (29), one can still obtain a reduced model via the balanced model reduction described in the Analysis section, as we do not assume any relationship among the parameters and initial conditions.

**Figure 3:**
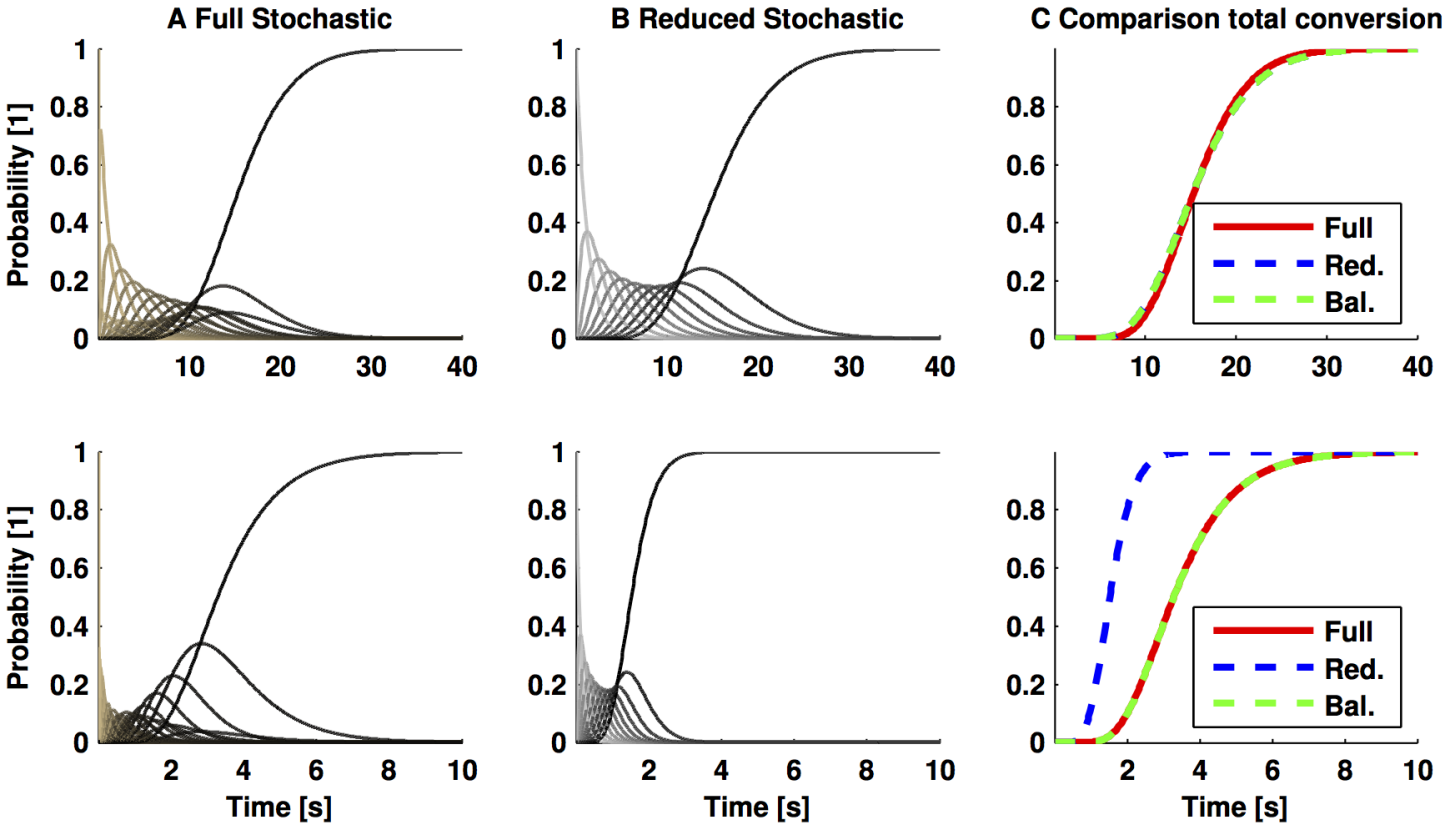
Validity of the Michaelis-Menten propensity as an approximation of a catalytic substrate conversion. Column **A** shows the simulation of the CME associated to (28), where each tread represents the probability of being in every state of the Markov chain; in turn, column **B** shows the solution of the CME of (29) by using the nonlinear propensity function (32); whereas, column **C** shows the probability of being in the last state of the Markov chain, which represents total conversion of the substrate to the product. This probability is obtained via the CME of the full stochastic model, by the CME considering the rapid equilibrium approximation, and by the approximated model to the CME via balanced realisation. The parameters used for obtaining the numerical solution are 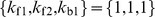 and 10 initial molecules of substrate. The only difference between the upper and lower panels is the number of enzymes considered: upper panels 1 molecule, whereas the lower panels, 10 molecules. Note that in the lower panel the rapid equilibrium approximation is not accurate, but the approximation via the balanced model truncation is close to the full model.

To exemplify the concepts above, we depict in Figure 3 a comparison of (**A**) the solution of the CME of (28) with propensities shown in Table 1; (**B**) the solution of the CME based on the rapid equilibrium approximation with the nonlinear propensity in (32); and (**C**) the solution of the reduced model described in the Analysis section for the last state of the Markov chain, which represents total conversion of the substrate to product. To simplify the notation, we consider a unitary volume of arbitrary units. The parameters values used are 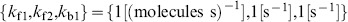 and 10 initial molecules of substrate.

The only difference between the upper and lower panels in Figure 3 is the number of initial molecules considered for the enzyme. In the upper panel we considered 1 molecule of the enzyme, hence condition (33) is fulfilled, and the rapid equilibrium approximation may be used to approximate the full model. Moreover, one can use the rapid equilibrium approximation to derive a reduced model via balanced realisation, as compared in the upper panel of Figure 3**C**. There, we approximated the model based on the rapid equilibrium approximation with 11 states by a reduced order model with 6 states; the 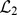 gain of the approximation error is less than 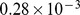, as given by (17).

In contrast, when we consider 10 molecules of enzyme initially, the CME derived from the rapid equilibrium approximation does not reproduce the dynamics of the full reaction network in (28), as depicted in the lower panels of Figure 3. We note, however, that we can still obtain a reduced model via balanced realisation that accurately approximates the dynamics of the full model (cfr. Figure 3**C** lower panel). There we approximated the full model with 66 states by a reduced model of 6 states, whose 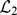 gain of the approximation error is less that 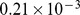.

Now we focus on the time required to simulate the CME and the time required to simulate the reduced order model. To compute the latter, we need to apply some state transformations to the CME (18) to derive a balanced realisation that can be further truncated. Once the reduced model is obtained, the time required for its numerical solution is significantly smaller compared to the time required for the numerical solution of the full CME.

To illustrate this reduction on the computational time, we obtained the CME of the reaction network (28) with an equal initial number of molecules for the substrate and enzyme and zero molecules for the rest of the species; later, we obtained the reduced order model via balanced realisation, which represents the state of total conversion of the substrate to the product; and finally we compared the time required for obtaining the numerical solution of the full CME (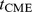) and the reduced model (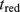) with the expression
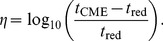
(34)


**Figure 4:**
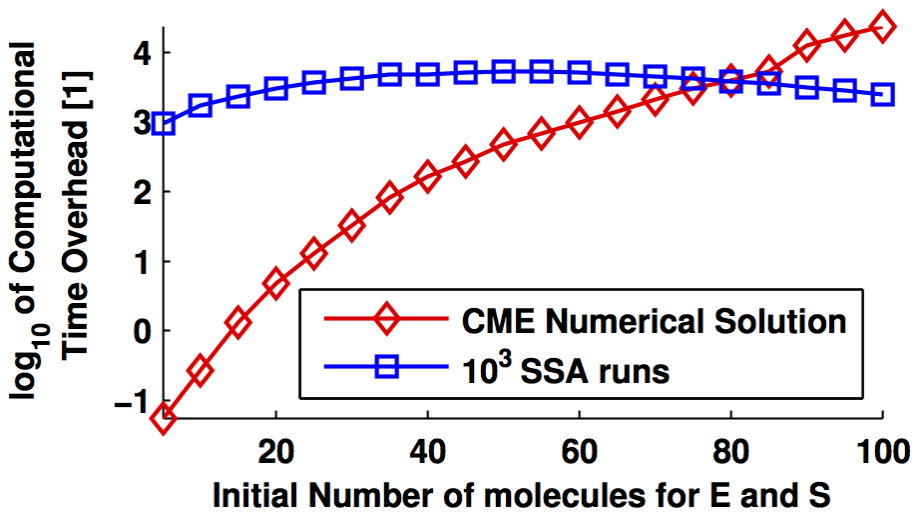
Computational time overhead of the numerical solution of CME and SSA with respect to numerical solution of reduced model. Computational time overhead, as given by (34), required to solve the full CME (diamonds) and to perform 10^3^ SSA runs (squares) as compared to the computational time required in seconds to simulate the reduced order model, as the initial number of molecules for *E* and *S* vary from 5 to 100. The parameters values used for simulation are identical to those of Figure 3.

We depict the results of this assessment in Figure 4. There we observe that as the number of molecules for 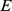 and 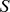 in the initial state increase, the savings on the computational time required to obtain the numerical solution of the lower-order model also increases. We note that for the comparison in (34) we did not account for the time required to obtain the reduced order model.

**Figure 5:**
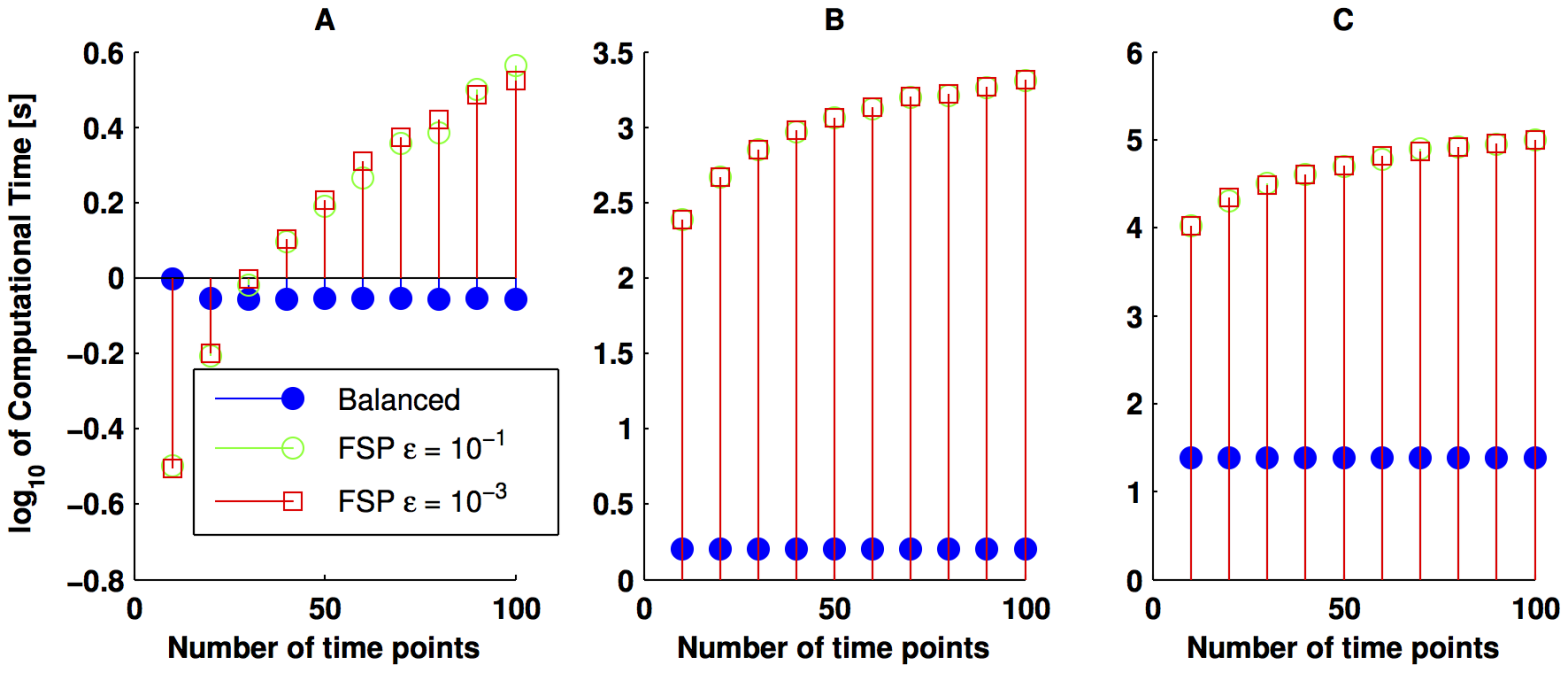
Computational time assessment of the FSP method vs balanced reduction approach. Comparison of the computational time required to obtain the reduced order model via balanced realisation (filled circle) and to obtain the approximative model via the FSP method (empty markers), with different, predefined error bounds (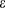). The reaction network analysed is (28). The parameters used for simulation are those of Figure 3. Panels 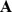, 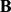, and 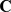 consider 10, 30, and 50 initial molecules for *E* and *S* and zero molecules for the rest of the species, respectively.

In turn, Figure 5 presents the comparison of the computational time required by *i*) the derivation of the reduced model via balanced realisation plus the simulation of the reduced model; and *ii*) the time required by the Finite State Projection (FSP) [31] for each time point. Of note, the FSP obtains an approximated probability vector with a desired error bound (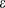) for *one specific time point*; hence, if one is interested in the transient response of the probability distribution, one has to run such an algorithm for every time step of interest. In contrast, once obtained the reduced model via balanced realisation, it is possible to use the lower-dimensional system for any number of time points and initial conditions. These results are summarised in Figure 5, where the panels 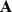, 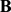, and 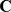 consider 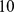, 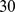, and 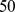 initial molecules for 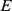 and 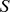, respectively, and zero molecules for the rest of the species. The remaining parameter values are identical to those of Figure 3.

Now, we assess the dimension of the state-space of the reduced-order models obtained by the FSP approach, an optimal finite state projection (OFSP) method ([32], Ch. 3), and the balanced model reduction method. The optimality of the second method refers to obtaining an approximation of the probability distributions for a specific time point with an error 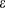 using the minimal number of states of the CME. The first step of this approach is to run any FSP algorithm such that the probability captured by this approach is 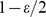. As a second step, this method proposes to keep the states with largest probabilities, such that the norm of the resulting probability vector is 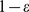. We refer the interested readers to ([32], Ch. 3) for a thorough explanation of this approach.

**Figure 6:**
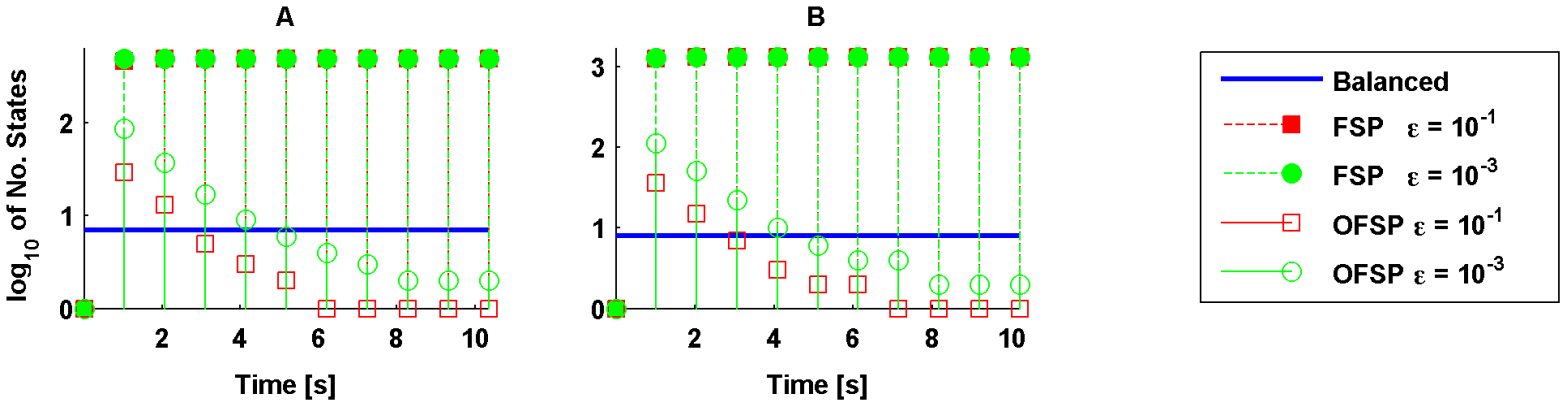
Number of states of different reduced-order models. By using different tolerances, the filled markers represent the number of states required by the FSP method, whereas the empty markers depict the number of states required by the OFSP. The continuous, blue line represents the number of states required by the reduced model via balanced realisation. The parameters values used for simulation are those used in Figure 3. Furthermore, panel **A** considers 30 molecules for *E* and *S* initially; whereas the initial condition for *E* and *S* in panel **B** is 50 molecules.

The results are summarised in Figure 6. This Figure depicts the number of states of the reduced model obtained via the balanced realisation with a continuous line to stress the fact that the model obtained by such an approach is valid for every time. In contrast, the methods based on finite projections require one run of the corresponding algorithms for each time-step of interest. Thus, in general, this may lead to larger computational loads, as suggested by the results in Figure 5.

To finalise, we note that for the FSP, the 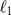 norm (sum of the absolute value of the entries of a vector) of the error bound is less than a predefined 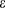 for the specific time points of interest (discrete signal), whereas the 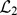 gain of the approximation error (continuous signal), obtained with the reduced model via balanced realisation, satisfies the bound given by (17). As the nature of both error signals is different, it is difficult to perform a direct comparison of the methods' accuracy. In the forthcoming section, we obtain a reduced order model that approximates the probability of having a certain range of 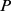 molecules.

### Probability for Ranges of Molecules Counts

Up to now, we have obtained reduced models that approximate the probability of being in one state of the Markov chain. In this section, we revisit the reaction network in (28) by obtaining the probability of having a certain number of molecules within predefined ranges, as done in applications such as gene expression [33].

Here we consider the following parameter definitions: 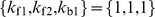, 100 initial molecules of substrate, 100 initial molecules of enzyme, and zero initial molecules for the rest of the species. By denoting the number of 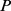 molecules with 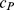, we can formulate our problem as approximating the following probabilities
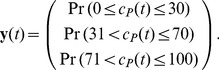
(35)


To derive the CME, one needs to obtain and label all the possible combinations of species molecular counts 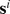 and organise them in the set 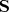 in (1). Then we have to evaluate the infinitesimal generator 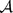 as in (12) with the corresponding reaction propensities of (28) (see Table 1).

In order to obtain an expression for 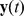, we need to define the matrix 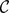 in (18b) so that the product of the first row of 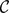 by the vector 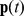 yield the sum of the probability of all the states 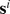 such that 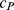 is within the range [0, 30]. The next two rows of 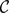 are defined in the same way, but accounting for the ranges 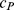 described in the second and third entries of (35).

**Figure 7:**
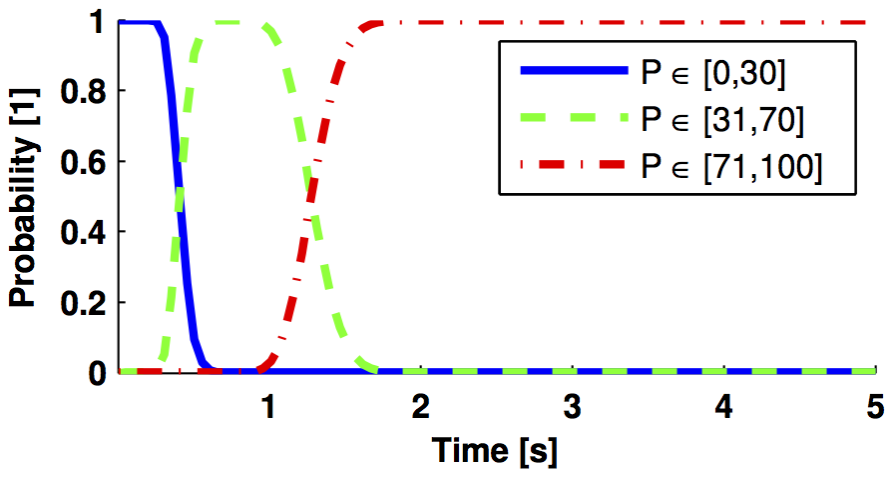
Marginal probability distributions for the reaction network (28). Probability of having a molecular count of *P* within a certain range, as obtained with the reduced order model. The parameters used for simulation are 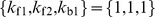, 100 initial molecules of substrate, 100 initial molecules of enzyme, and zero initial molecules for the remaining species.

The CME for this system, parameters, and initial number of molecules has 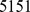 states. By applying the model reduction technique in the Analysis section, we can approximate the probabilities in (35) by a dynamical system with 16 states, whose output is depicted in Figure 7. The 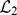 gain of the approximation error is less than 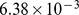, as estimated by (17).

### Brusselator

In contrast to the previous sections, here we consider the interaction of species which might exhibit an infinite number of population configurations. Namely, the Markov chain for this case exhibits an infinite number of possible states 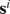. By analysing a large, yet truncated set of such configurations, we approximate the probability of the populations to be lower than a predefined threshold.

In particular, the system analysed is the Brusselator [34]. The reactions that compose this network are the following:
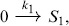
(36a)

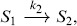
(36b)

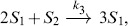
(36c)

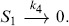
(36d)


In the deterministic set-up, the trajectories of the concentrations of 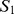 and 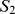 exhibit a limit cycle. When the system is considered to be stochastic, single trajectories of the SSA might preserve this oscillatory behaviour. However, when averaging over multiple trajectories of the SSA this oscillatory behaviour is in general lost, as not all the trajectories have the same frequency and phase of oscillation. Hence, the behaviour of the probability in time given by the solution of the CME will not present such limit cycle.

**Figure 8:**
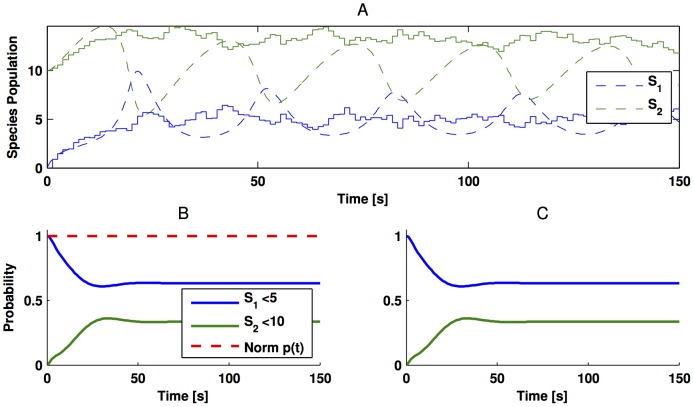
Trajectories of the Brusselator in (36). Panel 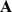 depicts the simulation of the deterministic (discontinuous line) and stochastic (continuous line) framework. For the stochastic approach, we averaged 100 trajectories of the SSA. In turn, Panel 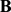 shows the numerical solution of the CME; whereas panel Panel 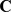 depicts the approximated numerical solution obtained via the reduced order model. In Panel 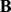 the red, discontinuous line represents the sum of all the states of the truncated Markov chain. The parameter values used for simulation are 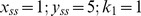 and 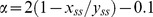; 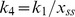; 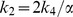; 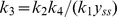.

Figure 8**A** presents the comparison between the numerical solution of the deterministic model and the average of 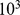 trajectories of the SSA. There we note that, although the deterministic trajectories present a sustained oscillation, the average of the SSA trajectories tends to a constant. This is confirmed by Figure 8**B**, where we depict the numerical solution of the CME for
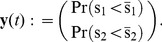
(37)


Here 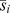, 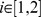 represents the steady state of the deterministic ODE. That is, each entry of 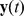 represents the probability of being below its steady state. Likewise, Figure 8**C** shows the approximation of (37) via the reduced model presented in this paper.

As mentioned earlier, the possible number of species population count in (36) is infinite as this is an open system subject to influx of 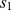. Hence, to obtain the results in Figure 8, we truncated the state space of the Markov chain to 3000 states. From this truncated Markov Chain, we obtained a reduced order model with only 10 states, whose 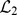 gain of the approximation error is less than 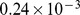. To confirm that this state-space truncation captures the support of the probability density function, we also tracked the sum of the probability for all the states. The red, discontinuous line in Figure 8**B** shows that this truncation includes all the probable states of the Markov chain.

## Discussion

In this paper we addressed the order reduction of the infinitesimal generator of a homogeneous, continuous-time, finite and discrete state-space Markov chain via the reduction of its balanced realisation. The application range of these dynamical systems is broad. Here, without loss of generality, we focus on its use on stochastic chemical reaction networks. In this context, the infinitesimal generator of the Markov chain that describes the probability of having a particular species molecular count is a large set of ODEs.

To reduce the order of the infinitesimal generator of a Markov chain, we used an alternative coordinate system to represent the chemical master equation (CME). This representation, denoted as Lyapunov balanced realisation, has the interesting property that the states of the transformed CME are organised in decreasing order, with respect to their impact on the probabilities of interest. Hence, an accurate approximation can be obtained, for example, by neglecting the last states of the Lyapunov balanced model, as discussed in the Analysis section. Although one may focus on particular states of the Markov chain, it is also possible to account for marginal probability distributions or even mean values, by properly defining the matrix 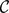 in (18b).

In many cases, only selected states of the Markov chain might be of practical relevance. For instance, this is the case when facing limited or inexact measurement data, or when only a few states are relevant for downstream signalling in biochemical reactions. Also, in imaging analysis of chemical reaction networks, obtaining the exact count of intracellular protein reporters might be challenging, due to limited resolution. Hence, the validation of the mathematical model that describes the process under observation should yield the probability of having a specific range of molecules count of the observed species.

We presented this procedure as a case study in the Results section, for a very simple reaction network. Even in such a simple case, the associated Markov chain has approximately 5000 distinct states of the system. This highlights how simulation of a system, even in the simplest cases, might imply a computationally intensive task. To alleviate such a burden, the model reduction via balanced realisation used in this paper yields lower-dimensional ODE sets, whose numerical solution might be several orders of magnitude quicker than the numerical solution of the original CME. Moreover, the method used to derive the lower dimensional model provides an upper bound on the approximation error, depending on the number of states neglected to derive the approximation.

In some cases, the processes required for deriving the reduced order model itself might take longer computational times, as compared to the mere simulation of the CME. Nevertheless, depending on the number of molecules of the system, the numerical solution of the reduced model might be obtained orders of magnitude faster, as shown in Figure 4. Hence, there will be real savings on the computational time when the reduced model is repeatedly utilised, for instance when adopting different initial probability distributions.

We would like to stress that to obtain a reduced order model, we have to fix kinetic parameters and to define which are the states of interest. Should we require to modify either of them, a new reduced model has to be derived. Likewise, all methods that require computational calculations, such as the FSP, SSA, and numerical solution of the CME will require numeric values for the parameters and, moreover, specific numerical values for the initial probability distribution. When either of them are modified, a new numerical solution has to be obtained. Additionally, the reduction and simulation of the CME might be orders of magnitude faster than the application of FSP-based methods, as suggested by an example analysed in the section Results.

Another possible use for the reduced model is to derive closed-form expressions of its solution (see [18], for instance), thereby avoiding the need for numerical solution of the reduced ODE set. When the number of states of the Markov chain to reduce is so large that using only one computer is unfeasible, we suggest the use of parallel algorithms to obtain the model reduction by truncation (see e.g. [25], [35]).

It is important to note that the reduced order model might lack some properties of the full model. For instance, the infinitesimal generator of the Markov chains studied here describes a positive system: the value of the probabilities will be always positive. However, the reduced order model obtained by truncation used in this paper will not, in general, preserve such a property. This implies that if most of the states of the balanced realisation are neglected to obtain the reduced model, there is a risk of having small, negative values for the approximated probabilities. An example of such phenomenon can be observed on the upper panels of Figure 2**A** and **B**. This evidences the existence of a trade-off on the order and the accuracy of the reduced-order model.

As a rule of thumb, a good approximation may be obtained by neglecting those states associated to Hankel singular values which are three orders of magnitudes smaller than the largest one. If the possibility of small, negative values for the probability cannot be afforded for the application of the reduced order model, there are other model order reduction methods that preserve the positivity of the original model, such as the recent works in [36]–[38]. However, it is equally important to note that these approaches are not generally applicable; are more time consuming; and have larger error bounds.

## Supporting Information

Appendix S1 **Two mathematical proofs.**
(PDF)
