# Supplementary material for: Order Reduction of the Chemical Master Equation via Balanced Realisation
Source: PLoS One. 2014 Aug 14;9(8):e103521. doi: 10.1371/journal.pone.0103521 (PMC4133211; doi:10.1371/journal.pone.0103521)
Supplement: Appendix S1 — Two mathematical proofs. (PDF) [file pone.0103521.s001.pdf]

# Supplementary information to “Order Reduction of the Chemical Master Equation via Balanced Realisation”

Fernando López-Caamal, Tatiana T. Marquez-Lago*
Integrative Systems Biology Unit, Okinawa Institute of Science and Technology, Kunigami, Okinawa, Japan

## 1 Proof of Nonpositive Eigenvalues of $\mathcal{A}$ in (8) of the Main Manuscript

In this section we prove that the eigenvalues of $\mathcal{A}$ in (8) have nonpositive real part. The cornerstone for this proof is the following theorem.

**Theorem 1.1** *[1, 6.1.1].*
*Let $\mathcal{A} \in \mathbb{R}^w$, let*

$$R'_i(\mathcal{A}) = \sum_{j=1, j\neq i}^{w} |a_{ji}|, \quad \forall\ i \in [1, w].$$

*Furthermore, consider the $w$ Geršgorin discs*

$$\{z \in \mathbb{C} : |z - a_{ii}| \leq R'_i(\mathcal{A})\}, \quad \forall\ i \in [1, w].$$

*Then, the eigenvalues of $\mathcal{A}$ are in the union of the Geršgorin discs*

$$G(\mathcal{A}) = \bigcup_{i=1}^{w} \{z \in \mathbb{C} : |z - a_{ii}| \leq R'_i(\mathcal{A})\}.$$

The theorem above says that the eigenvalues of $\mathcal{A}$ lay in the union of disks within the complex plane, each of which is centred on the point $a_{ii}$ and has a radius equal to the sum of the absolute value the entries of the $i^{th}$ column, except for the entry $a_{ii}$.

Now, for proving that the eigenvalues of $\mathcal{A}$ in (8) of the main manuscript are nonpositive, we note that (7) states $a_{ii} = -\sum_{j=1, j\neq i}^{w} a_{ij}$. Hence, all the Geršgorin discs are in the closed left-hand complex plane. Which imply that the real part of every eigenvalue of $\mathcal{A}$ in (8) must be nonpositive.

*Corresponding Author: Email tatiana.marquez@oist.jp

## 2 Derivation of the $\mathcal{L}_2$ error gain in (17) of the Main Manuscript

Here, we provide some definitions and the derivation of the approximation error bound (17) that arises from the model reduction via balanced realisation described in the section Analysis of the main manuscript. The material of this section is based on [3, 2].

First, to assess the size of the error of approximation, let us define the $\mathcal{L}_2$ norm of a real, time-dependent vector $\mathbf{u}(t)$ as

$$||\mathbf{u}||_{\mathcal{L}_2} := \left( \int_0^{\tau} \mathbf{u}^T(t)\mathbf{u}(t)\mathrm{d}t \right)^{1/2} .$$

When $\tau < \infty$, one obtains the norm of the truncated signal $\mathbf{u}(t)$. To increase readability, we will not explicitly show the upper limit of integration in the norm's subscript.

Now, in the frequency domain, the linear ODE (13) becomes the following algebraic equation

$$\mathbf{Y}(\xi) = \mathbf{G}(\xi)\mathbf{U}(\xi), \tag{S1}$$

where $\xi$ is the complex frequency variable that arises from the Laplace transform of (13), and

$$\mathbf{G}(\xi) := \mathbf{D} + \mathbf{C}\left(\xi\mathbf{I} - \mathbf{A}\right)^{-1}\mathbf{B}.$$

The complex matrix $\mathbf{G}(\xi)$ is denoted as the *transfer function* of the system (13) and characterises its input-output behaviour. The $\mathcal{H}_\infty$ norm of the complex matrix $\mathbf{G}(\xi)$ is defined as

$$||\mathbf{G}||_{\mathcal{H}_\infty} := \sup_{\mathrm{Re}(\xi)>0} \sqrt{\bar{\lambda}\left(\mathbf{G}^*(\xi)\mathbf{G}(\xi)\right)}.$$

Here $\bar{\lambda}(\circ)$ denotes the largest eigenvalue of the argument. In turn, the $\mathcal{H}_2$ norm of $\mathbf{G}(\xi)$, for analytic matrices on the open right half-plane, is

$$||\mathbf{U}||_{\mathcal{H}_2}^2 = \frac{1}{2\pi} \int_{-\infty}^{\infty} \mathrm{Trace}\left(\mathbf{Y}(j\omega)^*\mathbf{Y}(j\omega)\right) \mathrm{d}\omega.$$

When $\mathbf{U}(\xi)$ in (S1) belongs to the Banach space endowed with the norm $\mathcal{H}_2$, Theorem 4.4 in [3] states that

$$||\mathbf{Y}||_{\mathcal{H}_2}^2 = ||\mathbf{G}||_{\mathcal{H}_\infty}^2 ||\mathbf{U}||_{\mathcal{H}_2}^2. \tag{S2}$$

In order to relate the frequency-domain norms with the time-domain norms, we note that the Laplace transform used to obtain the transfer function of (13) in the main manuscript is an isomeric isomorphism between the $\mathcal{H}_2$ space in the frequency-domain and the $\mathcal{L}_2$ space in the time-domain.

Thus, from (S2), we can infer that

$$||\mathbf{y}||^2_{\mathcal{L}_2} = ||\mathbf{G}||^2_{\mathcal{H}_\infty}||\mathbf{u}||^2_{\mathcal{L}_2}. \tag{S3}$$

Now, we are ready to present the error bound due to the model-order reduction as presented in [2].

**Theorem 2.1** *[2, Th. 11.1] Let* $\mathbf{G}(\xi)$ *be a stable rational transfer function with Hankel singular values* $\sigma_1 \geq \sigma_2 \geq ... \geq \sigma_w$ *and let* $\mathbf{G}_{\text{red}}(\xi)$ *be obtained by truncating or residualising the balanced realization of* $\mathbf{G}(\xi)$ *to the first* $k$ *states. Then*

$$||\mathbf{G} - \mathbf{G}_{\text{red}}||_{\mathcal{H}_\infty} \leq 2 \sum_{i=k+1}^{w} \sigma_i. \tag{S4}$$

Hence, the relationship in (S3) implies

$$\frac{||\mathbf{y} - \mathbf{y}_{\text{red}}||_{\mathcal{L}_2}}{||\mathbf{u}||_{\mathcal{L}_2}} \leq 2 \sum_{i=k+1}^{w} \sigma_i. \tag{17}$$

# References


[1] R. A. Horn and C. R. Johnson. *Matrix analysis.* Cambridge university press, 2012.

[2] S. Skogestad and I. Postlethwaite. *Multivariable feedback control: analysis and design*, volume 2. Wiley New York, 2007.

[3] K. Zhou, J. C. Doyle, and Glover. *Robust and optimal control*, volume 40. Prentice Hall New Jersey, 1996.
